# Spatio-temporal models to determine association between *Campylobacter* cases and environment

**DOI:** 10.1093/ije/dyx217

**Published:** 2017-10-24

**Authors:** Roy A Sanderson, James A Maas, Alasdair P Blain, Russell Gorton, Jessica Ward, Sarah J O’Brien, Paul R Hunter, Stephen P Rushton

**Affiliations:** 1 Biological, Clinical and Environmental Systems Modelling Group, Newcastle University, Newcastle upon Tyne, UK; 2 Norwich Medical School, University of East Anglia, Norwich, UK; 3 Field Epidemiology Services North East, Public Health England, Newcastle upon Tyne, UK; 4 Institute of Infection and Global Health, University of Liverpool, Liverpool, UK

**Keywords:** *Campylobacter*, hydrology, livestock, rainfall, soils

## Abstract

**Background:**

Campylobacteriosis is a major cause of gastroenteritis in the UK, and although 70% of cases are associated with food sources, the remainder are probably associated with wider environmental exposure.

**Methods:**

In order to investigate wider environmental transmission, we conducted a spatio-temporal analysis of the association of human cases of *Campylobacter* in the Tyne catchment with weather, climate, hydrology and land use. A hydrological model was used to predict surface-water flow in the Tyne catchment over 5 years. We analysed associations between population-adjusted *Campylobacter* case rate and environmental factors hypothesized to be important in disease using a two-stage modelling framework. First, we investigated associations between temporal variation in case rate in relation to surface-water flow, temperature, evapotranspiration and rainfall, using linear mixed-effects models. Second, we used the random effects for the first model to quantify how spatial variation in static landscape features of soil and land use impacted on the likely differences between subcatchment associations of case rate with the temporal variables.

**Results:**

Population-adjusted *Campylobacter* case rates were associated with periods of high predicted surface-water flow, and during above average temperatures. Subcatchments with cattle on stagnogley soils, and to a lesser extent sheep plus cattle grazing, had higher *Campylobacter* case rates.

**Conclusions:**

Areas of stagnogley soils with mixed livestock grazing may be more vulnerable to both *Campylobacter* spread and exposure during periods of high rainfall, with resultant increased risk of human cases of the disease.

## Introduction

In the UK, *Campylobacter* is a major cause of gastroenteritis, and is thought to result in approximately 700 000 cases per annum, leading to health-associated costs in 2009 of over £50 million.^1^ The number of human cases of disease is strongly seasonal, with peaks in early summer (June) that vary regionally.^2^ Infection is mainly caused by consumption of contaminated chicken and beef, and chicken has been identified as a particular problem^3^ with the majority of samples bought at UK supermarkets found to be contaminated by *Campylobacter.*^4^ Although the majority of human *Campylobacter* cases can be linked to food consumption, between 30% and 50% of cases may be a result of infection from the wider environment.^5^ The survival and distribution of *Campylobacter* in the environment change with both space and time, and this will interact with how humans are exposed to the organism. However, given that the numbers of reported *Campylobacter* cases are dominated by infection through food, and eating behaviour changes seasonally^6^, this makes it harder to detect cases of infection from the wider environment. Seasonal variation in the prevalence of *Campylobacter* has not been detected in poultry^7^ or sheep,^8^ but the amounts of *Campylobacter* being shed by dairy cattle does change seasonally.^9^ To understand the epidemiology of *Campylobacter* therefore requires analyses that include spatial-temporal patterns, livestock management, meteorology and environmental conditions.

**Key Messages**

- *Campylobacter* is a major cause of gastroenteritis in the UK, with approximately 30% of cases associated with environmental contamination.
- We used hydrological and meteorological data in a temporal model, and livestock and soil maps in a spatial model, to assess potential environmental factors affecting human campylobacteriosis.
- Warm wet weather, during periods of high surface-water overland flow, combined with cattle grazing on stagnohumic gley soils, were associated with increased *Campylobacter* case rates.
- To understand *Campylobacter* case rates it is essential to measure the role of environmental factors such as meteorology, hydrology, livestock grazing and soil type.
- More information is needed on human behaviours, especially where and when visits are made to the countryside, that may affect the risk of exposure to *Campylobacter.*

Molecular epidemiological investigations suggest that the spring\early summer peak of *Campylobacter* infections may be largely due to environmental exposure.^2^ Complex pathways of primary and secondary interactions occur between *Campylobacter* reservoirs in soil, water, wild animals and livestock in the countryside,^10^^,^^11^ and whereas there is evidence for a wide distribution of *Campylobacter* in the environment, the health risks posed for humans remain unclear. Sequence type (ST) analyses of *Campylobacter* in Cheshire in North West England^12^ have indicated the particular importance of dairy cattle, and cattle-derived strains were most often isolated in humans (particularly the ST-61 complex). Cattle appear to have generally higher infection levels than sheep, at about 90% for herds and 55% for flocks.^3^^,^^13^*Campylobacter* deposited in faeces from individual sheep and cattle has been estimated at 10^2^ to 10^7^ colony-forming units/g,^9^ although this may be focused on a small number of ‘high-shedding’ animals within a herd.^3^

*Campylobacter* survival at any point location will depend on both local soil type and local weather conditions. Soil type is of particular importance^14^^,^^15^ as it affects soil moisture, chemistry and infiltration within the soil;^16^^,^^17^ some studies have indicated that *Campylobacter* is twice as common in clay compared with non-clay soils.^18^*Campylobacter* is microaerophilic and is extremely sensitive to desiccation in warm dry weather and UV radiation,^19^ and it survives better in the environment at temperatures less than 10°C compared with over 25°C ^18^^,^^20^ Some *Campylobacter* populations, however, also appear to have adaptive tolerance in the field to some environmental stresses.^20^ Wet conditions (due to soil type and/or weather) will therefore aid not only *Campylobacter* survival, but also increase its risk of being spread further during subsequent surface-water flow events. Human *Campylobacter* infection from environmental sources will be the product of two processes,: the presence of the pathogen and the mechanisms leading to human exposure. There remains considerable uncertainty about the role of different environmental factors in determining *Campylobacter* survival in the landscape, transmission between hosts and infection in humans. The pathogen is likely to be distributed in large quantities across the landscape through manure spreading, grazing livestock and transmission between domesticated animals and wildlife such as badgers, rabbits and wild birds.^21^^,^^22^

*Campylobacter* shed by livestock can be transported by surface and subsurface-water flows,^23^ and there is a large literature on predicting hydrological flows in landscapes and attempts to link this research with the distribution of pathogens.^24–26^ Most catchment-level landscape models of bacterial movement have focused on overland flow,^26^ although there have been recent attempts to produce models for small catchments which couple both surface and subsurface movement of bacterial pathogens.^25^^,^^27^ Quantifying the links between human disease and water flow is difficult because of differences in spatial and temporal scales: the cases of disease are comparatively infrequent, with delays between infection and reporting, and high-precision predictions of hydrological flow generally work best for small catchments.^28^ For large catchments such as that of the River Tyne, where spatio-temporal patterns in *Campylobacter* cases become more obvious, modelling surface flows becomes challenging, and the problems of integrating disease and environmental models operating at very different spatial and temporal scales increase.^29^ Storm events are likely to increase the amount of overland flow^30^^,^^31^ and are known to be important in the movement of other bacteria such as *Escherichia coli.*^30^ The frequency and intensity of these events are likely to increase with climate change, but their impacts on potential spread of *Campylobacter* are unclear.^32^

It is clear that any approach to investigate possible environmental factors associated with increased *Campylobacter* cases must incorporate the spatio-temporal dynamics of factors that might affect exposure, including weather conditions, catchment hydrology (especially surface-water flows that distribute the pathogen), soil types and livestock grazing patterns, while allowing for the vastly different scales of each process. The primary aim of this study was to improve our understanding of the impact and transmission pathways of environmentally acquired *Campylobacter* cases in the catchment of the River Tyne, and livestock land use, hydrology, soil conditions and meteorology, using a combined spatio-temporal statistical modelling approach. We hypothesize that the numbers of human cases of *Campylobacter* will be related to temporal variation in weather and hydrology (rainfall, run-off and temperature) which will impact on the distribution of *Campylobacter* in the environment, and that this will be moderated by spatial variation in livestock production, soil type and meteorology. The research had the following specific objectives:

- to quantify the association between occurrences of human cases of *Campylobacter* and temperature, rainfall and hydrological responses of the study catchment. We refer to this as our temporal model;
- and to investigate the association between the occurrence of *Campylobacter* cases and land use and soil after adjusting for the temporal variation in weather and hydrology. We refer to this as our spatial model.

**Figure 1:**
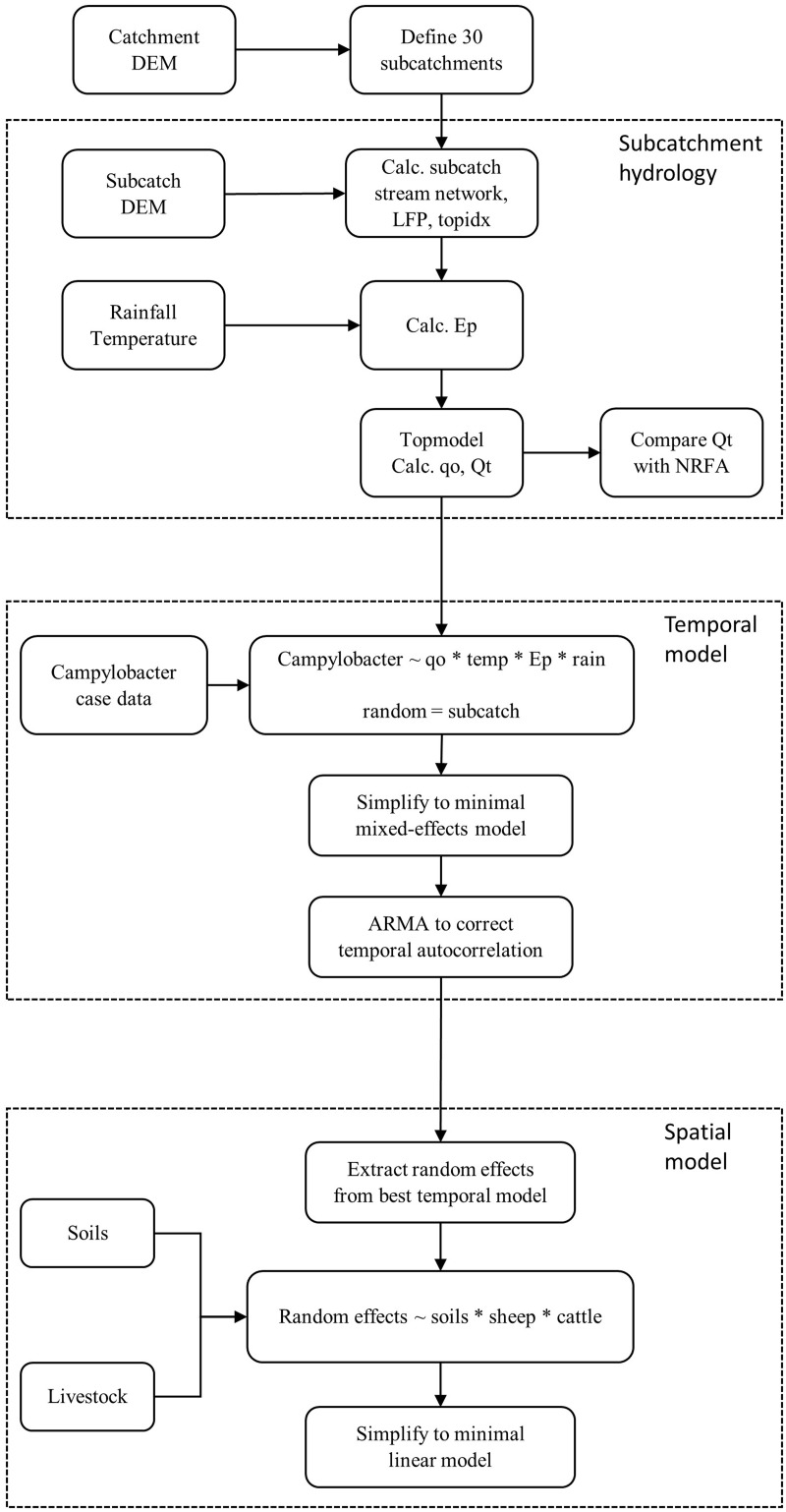
Flowchart to summarize procedures used to construct the Topmodel hydrological model, the temporal model of *Campylobacter* cases and spatial model to relate to soil type, sheep and cattle grazing. (Qt, total flow; qo, overland flow; Ep, evapotranspiration).

The Topmodel hydrological model^33^ was initially run for the study catchment and its predictions used as inputs into the temporal model. It investigates *Campylobacter* case rates in relation to short-term variation in the weather and hydrology across the range of subcatchments. The spatial model quantifies the effects of spatial variation in the underlying landscape (soil and livestock) of these subcatchments with case rates, having already been adjusted for temporal variation in weather and hydrology. The overall structure of the hydrological, temporal and spatial models is summarised in Figure 1.

## Methods

Analyses were undertaken using a combination of Unix shell-scripting to interface with the GRASS geographical information system,^34^ the Topmodel hydrological model^28^^,^^35^ and the R statistical package.^36^ One advantage of GRASS, as the geographical information system (GIS) for this type of analysis, is that it integrates well with R.^37^

### Hydrological model

#### Study area and division into subcatchments

**Figure 2:**
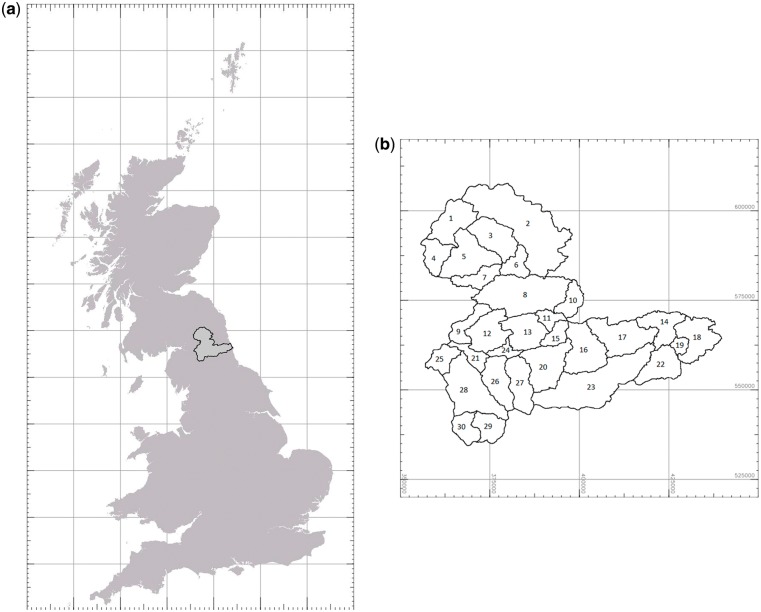
(a) Location of the Tyne catchment within the UK, with 100-km GB National Grid; and (b) division into 30 subcatchments, with 10-km grid, for use in the analyses.

The Tyne catchment is approximately 2944 km^2^ in North East England (Figure 2). The landscape is highly diverse, ranging from semi-natural wild moorland habitats dominated by upland plant species and sheep grazing (c 1000 km^2^) through lowland arable (c 275 km^2^) to the urban sprawl of Tyneside (c 220 km^2^). Kielder Valley, in the north-west of the catchment, contains Kielder Water, the largest reservoir in the UK at over 10 km^2^, and part of Kielder Forest (c 380 km^2^ coniferous plantation within the catchment). The catchment has an altitude range of 0 to 900 m, with average annual rainfall ranging from 630 mm to 1670 mm.

The Topmodel hydrological model^33^ was used to predict surface-water overland flow in the Tyne catchment from 1 January 2004 to 31 March 2009, using landscape topology and weather data as inputs. Topmodel breaks down a catchment into a series of subcatchments which are relatively homogeneous hydrologically (it is sometimes described as a ‘semi-distributed’ model). The subcatchments used to divide the main Tyne catchment were derived from the Ordnance Survey 50 -m raster^38^ map; the number of subcatchments to use is somewhat subjective, but has implications for subsequent analyses. If a large number of small subcatchments is created, there may be insufficient meteorological data within each subcatchment at sufficiently fine resolution to provide inputs for the hydrological models and subsequent analyses. In addition, there may be too few cases of human campylobacteriosis to analyse within small subcatchments. Conversely, if a small number of large subcatchments is created, the hydrological models will become more accurate, but there may be an inadequate number of subcatchments to provide sufficient statistical power to investigate spatial variation in soil type or livestock management effects. We created maps with 20, 30 and 40 subcatchments using the GRASS ‘r.watershed’ command, and undertook identical sets of analyses in each set. In practice, the results of the models were similar for all three sets of subcatchments; for brevity, we only report results from 30 subcatchments in detail in this paper.

#### Topmodel hydrological model 

The Topmodel rainfall-runoff model^33^ was used to predict surface-water overland flow at a daily time-step within each subcatchment. The two main assumptions which Topmodel uses to relate downslope flow from a point to discharge at the catchment outlet are that:

- the dynamics of the saturated zone are approximated by successive steady-state representations;
- and the hydraulic gradient of the saturated zone is approximated by the local surface topographic slope.

The model uses a relatively simple relationship between catchment storage and local water table depth, which can be related by the topographic index:^39^

(1) $$topidx=ln\left(\frac{a}{\tan\left(\beta \right)}\right)$$

where *a* = upslope contributing drainage area above a point and *β* = local slope angle. High values of *topidx* generally have large upstream contributing areas and\or shallow slopes such as at the base of hillsides or near streams; low values have small upslope-contributing areas and\or steep slopes.

A Linux bash script was stepped through for each subcatchment, using the appropriate digital elevation maps (DEM) and meteorological data. We used this DEM to derive stream networks in each subcatchment, rather than already published stream networks, to ensure maximal agreement between different inputs into Topmodel, especially measures of longest flow path and *topidx* values (see below). There were a few problems in accurately defining some stream networks, particularly on lowland or relatively flat areas towards the east of the Tyne catchment, or due to minor errors in the DEM, and these were resolved by running the relevant GRASS commands at a coarser 100 -m grid resolution. Daily meteorological data, provided by the UK Meteorological Office, were obtained for the study area from the British Atmospheric Data Centre (BADC) Meteorological Office Integrated Data Service (MIDAS) website, for the period 1 January 2004 to 31 March 2009.^40^^,^^41^ These data are provided in the form of -km grid resolution raster data. We calculated longest flowpath of a stream network within each subcatchment using the GRASS GIS ‘r.stream’ modules, in particular ‘r.stream.distance’ and ‘r.lfp’. The latter calculates the longest flowpath plus the cumulative drainage area upstream at points along the longest flowpath.^35^ Topmodel also requires input of the potential evapotranspiration (*Ep*), which we calculated based on daily temperature, rainfall and latitude for each subcatchment using the method of Xu and Singh.^42^

Topmodel was run via GRASS ‘r.topmodel’ using the default parameter sets, as the model primarily responds to topography and meteorology and is less sensitive to the exact parameter values used.^28^^,^^43^ Topmodel as implemented in GRASS ‘r.topmodel’ required three main inputs: first, a parameter file, which contained the default parameter sets, plus the distance along the longest flowpath and cumulative upstream area ratios in each subcatchment; second, the map in the subcatchment of topographic index values, *topidx,* calculated according to Eqn. 1; and third, a file containing rainfall and potential evapotranspiration over time. Our aim was to compare saturation overland flow (*qo*) predicted by Topmodel across different subcatchments, rather than predict exact overland flow values in any one subcatchment. Output files from the separate Topmodel runs in each subcatchment were automatically concatenated into a single file by the Unix bash script, for ease of use in subsequent analyses in R.

Topmodel outputs were compared with daily flow records from the UK National River Flow Archive (NRFA). The NRFA data do not contain overland flow values, so the most suitable comparison variable is total flow (*Qt*) which is also output from Topmodel. Nine NRFA gauging stations were operational in the Tyne catchment throughout the study period. Water flow in these generally encompassed flows from multiple upstream subcatchments used in our Topmodel runs (Table 1); therefore daily *Qt* values for upstream subcatchments were aggregated to coincide with the relevant NRFA areas. Strong annual patterns in both *Qt* and NRFA flow data were observed; therefore to avoid spurious correlation, each set of data was de-seasonalized using a standard sine-cosine harmonic model:

(2) y=α+β sin⁡(2πt)+γ cos⁡(2πt)+ɛ

where *y* = NRFA flow data or *Qt* Topmodel predictions, *α, β, γ* = estimated model coefficients, *t* = day of year/365.25 and *ɛ* = residual error.

**Table 1.** Summary of major soil types in Tyne catchment according to the Soil Survey of England and Wales (SSEW) classification (excluding urban areas)

Description	SSEW soil ID code	Area (ha)	Comments
Lithomorphic rankers	3.1	173	Shallow soils over bedrock
Brown calcareous earths	5.1	157	Agricultural soils, <300 m alt.
Brown earths	5.4	19325	Agricultural soils, <300 m alt.
Brown sands	5.5	1248	Agricultural soils, <300 m alt.
Brown alluvial soils	5.6	4872	Agricultural soils, <300 m alt.
Podzols	6.3	557	Well-drained, acidic soils
Stagnopodzols	6.5	9427	Upland, wet, peaty topsoil
Stagnogley soils	7.1	99096	Seasonally waterlogged, lowland
Stagnohumic gley soils	7.2	81467	Seasonally waterlogged, upland
Alluvial gley soils	8.1	582	Riverine-derived gley
Cambic gley soils	8.3	2300	Subsoil not clay-enriched
Disturbed soils	9.2	1514	Deep cultivation/mining/quarries
Raw peat soils	10.1	43987	Undrained, organic, acidic

Alt, altitude.

Two separate de-seasonalized models were created (one with NRFA flow data as the response, the other *Qt* Topmodel predictions). Cross-correlation functions (CCF)^44^ between the residual errors of these two models were then calculated for a range of lag-distances (days) to determine the overall correlation between the observations and predictions, and any time lag that might have occurred as a result of landscape characteristics.

### Temporal model of effects of overland flow, temperature, rainfall and evapotranspiration on *Campylobacter* cases in subcatchments

The aim of the temporal model was to investigate the temporal variation in the population-adjusted *Campylobacter* case rate in relation to monthly changes in environmental variables associated with weather and hydrology. This was achieved through linear mixed-effects models (LMMs) with population-adjusted *Campylobacter* case rate as the response, weather and hydrology as fixed effects and subcatchment as the random effect, using the R package ‘nlme’^45^ which can also account for temporal autocorrelation.

The number of reported cases of *Campylobacter* per month in each subcatchment was obtained from the Health Protection Agency in 2010 (now Public Health England); they were available for 63 months, from January 2004 to March 2009. The HPA data provide the six-figure UK residential postcode for each *Campylobacter* case, and these were converted into Ordnance Survey GB National Grid eastings and northings and imported into the GIS. Cases were then overlaid onto the map of the subcatchments. The *Campylobacter* case data were log-transformed (corrected by log-transformed population size in each sub-catchment), to create a ‘population-adjusted *Campylobacter* case rate’ in each subcatchment per month. Topmodel outputs were daily; therefore the mean monthly temperature, rainfall, potential evapotranspiration (*Ep*) and saturated overland flow (*qo*) were calculated before comparison with the *Campylobacter* case data.

A standard mixed-effects model^45^ can be expressed in matrix formulation as:

(3) $$y_{i}=X_{i}\beta +Z_{i}b_{i}+\in_{i}$$

(4) $$b_{i}∼N_{q}\left(0,\Psi \right)$$

(5) $${ɛ}_{i}∼N_{ni}\left(0,\sigma^{2}\Lambda_{i}\right)$$

where *y_i_* = *n*_i_ x 1 vector of observations in *i*’th group, *X_i_* = *n*_i_ x 1 model matrix of fixed-effects regressors for observations in group *I*, *β* = *p* x 1 vector of fixed-effects coefficients, *Z_i_* = *n_i_* x *q* matrix of regressors for random effects for observations in group *I*, *b_i_* = *p* x 1 vector of random effects for group *I, ɛ_i_* = *n_i_* x 1 vector of errors for observations in each group, Ψ = *q* x *q* covariance matrix for random effects and *σ^2^*Λ = *n_i_* x *n_i_* covariance matrix for errors in group *i.*

This formulation gives considerable flexibility in the structure of mixed-effects models. In our study, we used subcatchment as the grouping variable (hence index *i* varied from 1 to 30). We did not detect evidence of long-term increases or decreases in the population-adjusted *Campylobacter* case rate during the study period; therefore a random-intercepts, fixed-slopes model was fitted. Initially overland flow, temperature, evapotranspiration and rain were used as predictors, plus all interaction terms, to account for potential collinearity between predictor variables. Non-significant interaction terms were sequentially removed, and where necessary main effects, and each simplified model compared with the previous one. Non-significant main effects were retained in the final model if they were included in a significant interaction term. Model fitting was done via maximum likelihood (ML) rather than the default restricted maximum likelihood (REML), because the fixed effects changed with each model.^46^ When comparing models, those with a lower Bayesian Information Criterion (BIC) were selected.

After identifying the best linear mixed-effects model, autocorrelation function (ACF) plots were constructed to check for evidence of temporal autocorrelation in the residuals. Initial modelling efforts were focused on identification of the best predictors (i.e. the fixed effects), and then improving these models to account for any temporal autocorrelation.^47^^,^^48^ Autoregressive (AR) moving average (MA) correlation terms were added, testing over a range daily lags for p (autoregressive) and q (moving average) between 0 and 3 to find the optimum values to correct for temporal autocorrelation.^49^^,^^50^ This approach has the advantage of exploring all correction options from pure autoregressive (p > 0 and q = 0), pure moving average (p = 0 and q > 0) or joint autoregressive moving-average (ARMA; p > 0 and q > 0), until the optimal correction is identified, by altering the structure of *Λ_i_* in the covariance matrix in Eqn. 5 above.

After identification of an appropriate AR, MA or ARMA correlation function (and confirmation of improvement in model fit via BIC and the ACF plots), the random effects (i.e. for each subcatchment) from this model were used as the response variable in the subcatchment analyses as described below.

### Spatial model of soil type, sheep and cattle stocking rates on *Campylobacter* cases

The random effects from the best temporal model quantify differences between the subcatchments in population-adjusted *Campylobacter* case rates which are not explained by the hydrology, temperature, evapotranspiration or rainfall. These spatial differences between the subcatchments might be due to other environmental factors, in particular soil type and livestock grazing.

Soil data from the Soil Survey of England and Wales (SSEW) maps for northern England^51^ at 100 -m grid resolution were analysed at the level of the soil group in the SSEW classification. Different soil groups show strong collinearity, and it was not practical to include all the soil groups (plus interactions with livestock) as explanatory variables in the subcatchment models. Therefore, to identify the main patterns of variation in the composition of SSEW soil groups across the subcatchments, the matrix of subcatchments by soil groups was initially analysed using principal components analysis (PCA). The subcatchments by soil groups matrix was Hellinger-transformed^52^ before the PCA analysis, as the total areas of each soil group within a subcatchment were non-independent. The soil type(s) with the highest correlations with PC1 (and if necessary PC2) were used as predictors in the linear models.

Data on the numbers of livestock derived from the Defra Agricultural Census (2010); these were obtained at 2-km grid resolution.^53^^,^^54^ Total numbers of sheep/km^-2^ and cattle/km^-2^ were calculated within GRASS for each subcatchment. There is evidence that sheep and cattle may play slightly different roles in the transmission of *Campylobacter* to humans;^3^ therefore they were used as separate predictor variables in the subcatchment analyses.

The linear models used the random effects from the temporal analyses as the response, with explanatory variables of sheep/km^-2^, cattle/km^-2^, and the soil type(s) most characteristic of variation between the subcatchments. All main effects and higher-level interactions were initially fitted to the full linear model, which was simplified until a minimal model with lowest BIC was identified.

## Results

### Hydrological model

#### Characteristics of subcatchments

**Figure 3:**
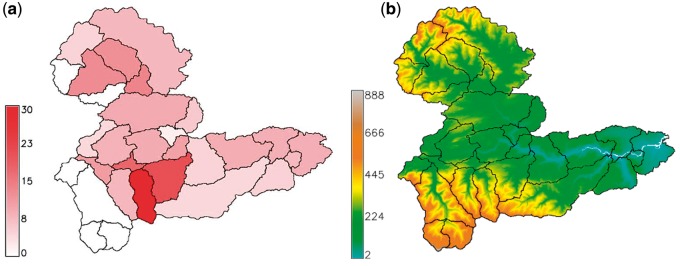
(a) Total number of *Campylobacter* cases per 1000 population in each subcatchment over whole study period; (b) elevation above sea level in metres.

The distributions of the 30 subcatchments are summarized in Figure 2b. The geographical extent of some subcatchments was unchanged irrespective of the number of subcatchments used to subdivide the whole Tyne catchment. These were generally subcatchments in the upland reaches of the Tyne catchment, where the topography was such that there was considerable difference in elevation from river valley to mountain tops, with the result that subcatchments could be clearly defined hydrologically, e.g. the Rede Valley, West and East Allendales: subcatchments 2, 26 and 27, respectively. In contrast, where the topography was flatter, there was more variation in subcatchment area and shape depending on whether 20, 30 or 40 subcatchments were used, e.g. subcatchments 14, 18, 19 and 22 around Tyneside. Highest *Campylobacter* case rates per head of population were in the Allendales and east of the catchment near Tyneside (Figure 3a). Irrespective of the number of subcatchments used, those in the north-west and south-west of the Tyne catchment were predominantly upland, with higher livestock numbers, compared with the lowland and urbanized eastern end of the catchment (Figure 3b). Soils in the Tyne catchment as a whole are dominated by gley soils with a high clay content, raw peats in the uplands and brown earths on some of the better low-altitude agricultural land (Table 1).

#### Topmodel hydrological model

**Figure 4:**
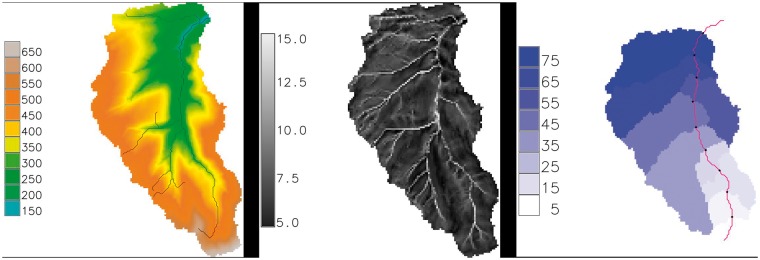
Example Topmodel outputs for River West Allen, subcatchment 26. (a) Elevation (metres) and predicted stream network; (b) topographic index scores *topidx*; (c) longest flow path (solid continuous line) and cumulative drainage basins (drained areas upstream km^2^).

Temperature and rainfall showed strong cyclical patterns on an annual basis, as might be expected, with knock-on effects on predicted evapotranspiration across the whole Tyne region. The saturated overland flow, *qo*, was less predictable, with large overland flow predicted by Topmodel during the winter of 2006–07. This was associated with a period of intense rainfall, but there were considerable variations between subcatchments in predicted overland flow as a result of between-subcatchment variation in temperature, rainfall and topography. For example, peak overland flow was very high in the south-west of the Tyne catchment in winter 2006–07, particularly around the River West Allen and East Allen, and River South Tyne near Featherstone (subcatchments 26, 27 and 28, respectively, in Figure 2b). In contrast, there was less evidence of any major change in overland flow in the north-west of the catchment during this period, even though this area also experienced higher rainfall. These are subcatchments 1 to 6, around the River Rede, Kielder Valley (including Kielder Water reservoir) and Tarset Burn. Although the north-west is also upland, it has fewer steep-sided valleys than the south-west, which accounts for the lower overland flow. Figure 4 shows more detailed Topmodel outputs for subcatchment 26 (River West Allen): the predicted stream network (Figure 4a); the *topidx* index, showing higher values near streams (Figure 4b); and the longest flowpath and cumulative drainage basins (Figure 4c).

De-seasonalized NRFA flow data were significantly positively cross-correlated with predicted Topmodel Qt estimates at all nine NRFA gauging stations (Table 1), the majority with a CCF of greater than 0.40 and median maximum CCF at a lag of 4 days. CCF values were lowest for the River Derwent (NRFA station 23007) and Team Valley (NRFA station 23017), both of which are lowland catchments that only partially encompass some of the subcatchments we defined for use in Topmodel and include relatively large urban areas. The highest time lag was 7 days recorded for the North Tyne (NRFA station 23022), but the NRFA warn that flow records from this gauging station are strongly affected by Kielder Reservoir, which is important for human-induced water discharge.

### Temporal model of effects of overland flow, temperature, rainfall and evapotranspiration on *Campylobacter* cases

The population-adjusted case rate was most strongly associated with overland surface-water flow, temperature and the interactions between overland flow and temperature, and between evapotranspiration and rain (Table 3), with case rates predicted to be higher with increased overland flow and in warm weather conditions. There was a spring peak in *Campylobacter* cases every year in the most densely populated subcatchments around Newcastle and Tynemouth (numbers 14, 17, 18, 19 and 22; Figure 2), but seasonal patterns were less consistent across all years of the study in the other subcatchments.

The minimal linear mixed-effects model showed strong residual autocorrelation, evident at lags 2 and 3 in particular, with an optimum ARMA model at lag p = 3 and moving average q = 2. The need for ARMA models could simply reflect time delays between *Campylobacter* infection and sample date, since the onset of disease post-exposure is variable (see Discussion). Examination of residuals indicated that the model fitted best to the catchments in the north-west of the Tyne catchment, and most poorly in the south and east of the catchment, especially some of the flatter, lowland urban areas, where it is possible that subcatchment area and extents were less accurately defined, or where the epidemiology of the disease was different.

### Spatial model of soil type, sheep and cattle stocking rates on *Campylobacter* cases

Over 60% of the variation in the composition of SSEW soil groups within each subcatchment was explained by the first PCA axis, which represented a trend from stagnohumic gleys and raw peats through to brown earths and stagnogleys. PCA axis 1 was most strongly positively correlated with the stagnogleys (r = 0.906, *P* < 0.0001); therefore the area of stagnogley in each subcatchment was used as an explanatory variable in subsequent analyses. In contrast, PCA axis 2 only explained 15% of the variation in SSEW soil composition, was not clearly associated with major soil types and was therefore omitted from subsequent analyses.

**Figure 5:**
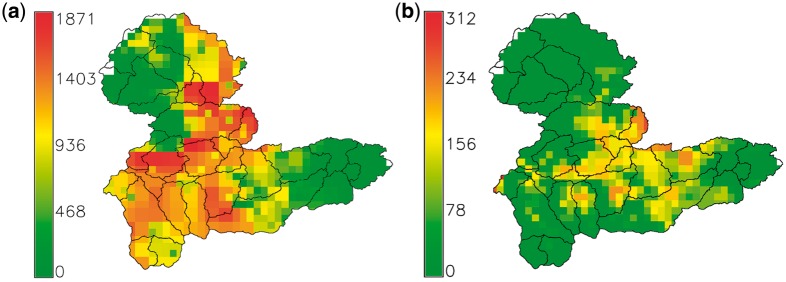
(a) Sheep and (b) cattle grazing in the Tyne catchment based on the DEFRA June 2010 Agricultural Census at 2-km raster scale; units are animals km^2^.

**Figure 6:**
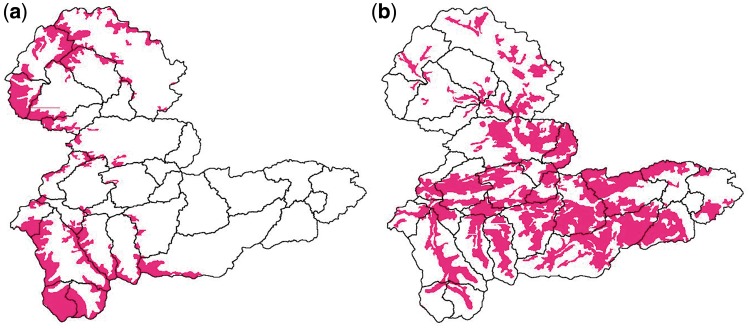
Soil Survey of England and Wales maps of: (a) raw peat soils and (b) stagnogley soils.

We assumed that the random effects from the best temporal model described above quantified the unexplained spatial variation across different subcatchments in the population-adjusted *Campylobacter* case rate. The subcatchment (linear) models used these random effects as response variables, to determine which environmental variables at the subcatchment level were most associated with deviations from the ‘average’ effect explained by the fixed effects of the temporal model. The amount of stagnogley, cattle density and interactions between the stagnogleys x cattle and sheep x cattle, were found to be the most important environmental variables of the random intercepts in the best subcatchment model (F_5,24_ = 17.39, Adj-R^2^ = 0.739, *P* < 0.0001; Table 4). The signs on the estimated coefficient values in Table 4 indicate that deviations from the predicted case rate from the temporal model were negative for individual subcatchments with larger areas of stagnogley (Figure 6b) and areas of higher numbers of cattle (mid-altitude: Figure 5b). However, the positive cattle x stagnogley interaction (Table 4; t = 2.334, *P* = 0.0283) suggests that in those subcatchments that contained large cattle numbers grazing stagnogley soils, the temporal model under-predicted *Campylobacter* case rate. Sheep grazing without cattle was mainly associated with the uplands (excluding the north-west around Kielder Forest), but the positive cattle x sheep interaction (Table 4; t = 2.958, *P* = 0.0069) suggests higher *Campylobacter* case rates in those (predominantly lower altitude) subcatchments which contained both sheep and cattle grazing.

## Discussion

This study has identified associations between population-adjusted *Campylobacter* cases rates and landscape hydrology, land use and meteorology. Meteorological conditions were important over the 63-month study period, with higher *Campylobacter* case rates associated with higher temperatures periods of high surface-water overland flow. Subcatchment hydrology was affected by both the weather conditions and topology of each subcatchment. Periods of high surface-water overland flow, in those subcatchments where the topography resulted in increased flow rates, were also associated with higher population-adjusted *Campylobacter* case rates. Within each subcatchment, stagnogley soils were associated with lower risks of *Campylobacter*, except in those areas where overall livestock density was high on stagnogleys, when the converse was true. When farm animal grazing type was broken down separately for sheep and cattle; the density of cows grazing on stagnogley soils was an important risk factor, as were larger densities of both sheep and cattle within a subcatchment.

The temporal model indicates that *Campylobacter* cases were higher during periods of high overland flow. Storm events are known to result in more rapid surface-water transport of bacterial pathogens,^55^^,^^56^ particularly when soils are already saturated. The amount of transport from livestock sources onto adjacent fields, paths and roads will depend partly on the land management, drainage ditches, small scale topography etc. Mole drains will increase the amount of subsurface transport.^23^ Runoff is generally lower on soils with a large soil pore structure, such as peats\stagnohumic gleys than more compacted clay soils, such as stagnogleys. Our use of Topmodel does not explicitly attempt to model the effects of soil type, land management or livestock management, but our results nevertheless indicate that over the 63-month study period, high overland flow was associated with increased *Campylobacter* cases. Note that the accuracy of the Topmodel predictions when compared with the NRFA data was unaffected by the predominant soil type in the relevant subcatchments (Table 2); predictions were least accurate in urbanized or flat lowland subcatchments.

**Table 2.** Comparison of National Rivers Authority (NRFA) daily flow rates and predicted Topmodel Qt (saturated and overland flow). Cross-correlation functions were calculated on the residuals from the annually de-seasonalized models for NRFA and Qt estimates described in Eqn. 1. The table indicates the maximum CCF values calculated, and the lag (in days) for this maximum. NRFA gauging stations usually measured rivers that encompassed multiple upstream Topmodel subcatchments, and data from the latter were aggregated before comparison

NRFA station name	NRFA code	Max. CCF	Lag (days)	Upstream Topmodel subcatchments
River Tyne, Benwell	23001	0.528	4	1–13, 15, 20, 21, 24–30
North Tyne, Reaverhill	23003	0.522	4	1–8
South Tyne, Haydon Bridge	23004	0.463	4	9, 12, 21, 24, 25, 26–30
South Tyne, Featherstone	23006	0.353	4	25, 28–30
River Derwent	23007	0.184	4	23
River Rede	23008	0.429	2	2
Kielder Burn	23011	0.409	3	1
Team Valley	23017	0.177	1	14, 19, 22
North Tyne, Uglydubb	23022	0.365	7	1, 4, 5

*Campylobacter* does not survive well with drying, and we originally assumed that there would be a negative effect of temperature on *Campylobacter* case rates; in practice the reverse was found (Table 3), with temperature and the temperature x evapotranspiration interaction associated with higher number of cases. There are several possible explanations for this apparent anomaly. First, some strains of *C. jejuni* are more resistant to oxidative stresses associated with drought and higher temperatures.^57^ The markers that encode for the genes thought to be associated with oxidative stress resistance are more common in strains from grazing livestock^5^ and have been detected at higher frequencies in grazed areas. Second, increased human *Campylobacter* case rates during the summer may simply reflect greater outdoor activity and exposure of local residents, transmission by wildlife and use of barbeques with a knock-on increased risk of consumption of partially cooked meat (although widespread barbeque use in remote, sheep-rearing, high rainfall areas in the south-west of the Tyne catchment where cases were highest, seems unlikely!). A spring peak in *Campylobacter* cases was observed across all years in the urban conurbation to the east of the catchment, and to a lesser extent in some, but not all, years in the more rural parts of the west and south-west of the catchment. Again, human behaviour may play a role in this peak, with larger number of visits to the countryside in the spring.

**Table 3.** Summary of results of best temporal linear mixed-effects model, with population-adjusted *Campylobacter* case rates per month as the response, and subcatchment number as the random effect. *qo* is saturated surface-water overland flow from Topmodel; *Ep* is potential evapotranspiration. Standard deviation of residual random effects = 0.3104; log-likelihood = −496.95; BIC = 1099.51

Coefficient	Value	SE	t-value	*P*-value
Intercept	−6.7822	0.3669	−18.484	<0.0001
*qo*	0.0802	0.0308	2.609	0.0092
Temperature	0.0702	0.0223	3.151	0.0017
*Ep*	0.0105	0.0217	0.484	0.6283
Rain	0.0102	0.0095	1.077	0.2819
*qo* x temperature	0.0776	0.0294	2.638	0.0084
*Ep* x rain	−0.0204	0.0091	−2.239	0.0253

SE, standard error.

The subcatchment analyses provide insights into spatial, i.e. subcatchment-level, processes that affect *Campylobacter* case rates, after having adjusted for the weather-related temporal changes over the study period. Stagnogleys are clay soils which have a relatively impervious subsurface horizon and a distinct topsoil, and are prone to waterlogging during periods of heavy rain.^58^ The negative main-effect coefficient for stagnogleys (Table 4; coefficient = -7.3675; *P* < 0.0001) indicates that *Campylobacter* cases were generally lower in those subcatchments, but only if the effects of livestock grazing are ignored (see later). Conversely, raw peats and stagnohumic gley were strongly negatively correlated with soil PCA Axis 1; if either are used instead of stagnogley as the soil predictor in the subcatchment analyses, they are associated with increased *Campylobacter* cases. Soil-borne pathogens survive better in a more open soil matrix that allows increased penetration into the soil and that contains more organic matter, with reduced desiccation during dry weather,^15^ such as raw peats and stagnohumic gley. Whereas published data on *Campylobacter* survival in stagnogleys are not available, survival would be expected to be lower due to the greater tendency of stagnogleys to surface desiccation in dry weather and the lower organic matter content. Stagnogleys are also more productive than stagnohumic gley and peat soils, and probably not subject to human access as frequently because they are more intensively managed.

**Table 4.** Summary of results of best spatial (subcatchment) model of the effects of soil type and livestock management, using the random effects from the best linear mixed-effects model as the response. F_5,24_ = 17.39; *P* < 0.0001; Adj-R^2^ = 0.739

Coefficient	Value	SE	t-value	*P*-value
Intercept	4.7730	0.6876	6.942	<0.0001
Stagnogley	−7.3675	1.0718	−6.874	<0.0001
Sheep km^−2^	−0.0014	0.0023	−0.619	0.5416
Cows km^−2^	−0.2618	0.0794	−3.299	0.0030
Stagnogley x cows km^−2^	0.2089	0.0895	2.334	0.0283
Sheep km^−2^ x cows km^−2^	0.0004	0.0001	2.958	0.0069

SE, standard error.

Subcatchments with high numbers of cattle were associated with decreased *Campylobacter* case rates, in contrast to the findings of most previous studies ^3^. Most cattle grazing in the Tyne catchment occurs in lowland areas immediately to the west of the main Newcastle\Gateshead conurbation (Figure 5b; subcatchments 10, 11, 13, 15, 16, 17 and 23). The decrease in *Campylobacter* case rates with cattle may result from people being less likely to cross fields stocked by cattle, or avoidance of slurry-treated areas and dung-pats. In addition, the risk of human infection within cattle-grazed fields may depend on the number of ‘high-shedding’ animals within the herd, rather than the average proportion of individual cows infected.^3^

Stagnogley soils are vulnerable to poaching by livestock, especially in wet weather,^59^ and this can be exacerbated by spatial clustering of livestock within fields, especially around water troughs, feeding areas etc. Poaching will lead to reduced infiltration of water into the soil, but will lead to increased surface-water flow.^60^ Higher rates of *Campylobacter* contamination have been recorded on clay soils such as stagnogleys.^18^ Our results accord with these earlier studies,^59^^,^^60^ with cattle grazing on stagnogley soils being associated with higher *Campylobacter* case rates (Table 4; stagnogley by cattle interaction). Higher *Campylobacter* case rates also occurred in subcatchments with both sheep and cattle (Table 4; sheep x cattle interaction). Whereas both sheep and cattle are known to be excretors of *Campylobacter*,^61^ our results suggest that cattle are more important (Table 4). Run-off water from sheep and cattle-grazed agricultural land, eventually entering the groundwater drinking supply, has also been identified as a source of human *Campylobacter* infections in France.^62^

There have been few attempts to investigate pathogen spread at the catchment scale, with some of the most detailed single studies being undertaken in Australia and New Zealand^25^^,^^27^ under different climatic and agricultural systems from those in the UK. We have used a standard hydrological model that is not excessive in either its input data requirements or CPU run time, to understand temporal changes in meteorological conditions, in combination with surface-water flow predicted from the hydrological model, to understand the changes in *Campylobacter* cases between 2004 and 2009. Although we did not have sufficient data to undertake more advanced fully integrated spatio-temporal analyses,^63^ the use of outputs from separate temporal analyses as inputs into subcatchment analyses has allowed us to investigate the relationships between livestock management, soil type and human *Campylobacter* cases. It is nevertheless clear that the environment has a major role, but the main limitation of this study was that it was not possible to quantify the relative importance of environmental versus food-borne sources of infection. Another challenge is the delay between infection and reporting of cases, which may partly explain the need to use ARMA models; delays from onset of infection to receipt of specimen are on average 5 days in England and Wales^6^ but is typically 16 days in Scotland,^6^ based on data collected from 1989 to 1999. Nevertheless, our calculated lag times are slightly less than might be expected a priori. In *Campylobacter* the incubation period is usually 2–5 days before symptoms arise; and in our data where a doctor could provide an onset date, the median was 4 days, giving an overall delay between infection and specimen of 6 to 9 days. This compares lags of to 2 to 3 days for our ARMA model. The types of analyses we describe would become much more powerful, and useful for public health, if we had had data on the strain types (ST) of the individual human *Campylobacter* infections, plus samples of *Campylobacter* taken from the countryside and livestock. Powerful Bayesian methods have recently been developed to source-attribute models, using ST data.^64^ This would permit a formal link of source to humans, particularly as our results suggest the infection sources change over time and that the risks to human health also depend on human behaviour and activity in the wider countryside.

## Funding

This work was supported by the Medical Research Council Grant, the Natural Environment Research Council, the Economic & Social Research Council, the Biotechnology & Biological Sciences Research Council and the Food Standards Agency through the Environmental & Social Ecology of Human Infectious Diseases Initiative (ENIGMA Consortium—study of *Campylobacter* project: grant reference G1100799/1).
